# Numerical Simulations of Calcium Sulphate Scaling in Full-Scale Brackish Water Reverse Osmosis Pressure Vessels Using Computational Fluid Dynamics

**DOI:** 10.3390/membranes11070521

**Published:** 2021-07-11

**Authors:** Weidong Mao, Xiang Zou, Zhongquan Guo, Sui Sun, Sai Ma, Shunzhi Lyv, Yan Xiao, Xinxiang Ji, Yuan Wang

**Affiliations:** 1 China Coal Technology & Engineering Group Hangzhou Research Institute Co., Ltd., Hangzhou 311201, China; w.d.mao@163.com (W.M.); gzq163@163.com (Z.G.); marseilles928@163.com (S.M.); hoho-xiao@163.com (Y.X.); 2 UNSW Centre for Transformational Environmental Technologies, Yixing 214200, China; xiang.zou@unswctet.com (X.Z.); sui.sui@unswctet.com (S.S.); shunzhi.lv@unswctet.com (S.L.); xinxiang.ji@student.unsw.edu.au (X.J.); 3 School of Civil & Environmental Engineering, UNSW Sydney, Sydney 2052, Australia

**Keywords:** spiral-wound module, reverse osmosis, RO, computational fluid dynamics, CFD, desalination, coal mine water, sparingly soluble salts, calcium sulphate, zero liquid discharge

## Abstract

Coal mine waters often have high salinity, hardness and alkalinity. The treatment of coal mine water requires careful management of multi-stage reverse osmosis (RO) systems to achieve effective recovery of water for domestic reuse, as well as zero liquid discharge to minimise the impact to the local environment. Design of RO systems for coal mine water treatment has been limited to the use of commercial design packages provided by membrane manufacturers, which do not provide insights into the impact of operating parameters such as feedwater salinity, concentrations of sparingly soluble salts, feed pressure and their interactions with different RO modules on the fouling/scaling potential of RO membranes. This also restricts the use of novel RO products and the delivery of an optimum design based on real needs. In this work, a mathematical model was developed to simulate a standard brackish water RO pressure vessel consisting six full-size RO membrane elements, using computational fluid dynamics (CFD). The model can be used to predict the permeate flowrate, water recovery levels, as well as the spatial information of the accumulation and scaling potential of sparingly soluble salts on the membrane surface. The results obtained from the model showed good agreement with the results obtained from the commercial RO design software WAVE. The CFD model was then used to predict the scaling threshold on various positions of a full-scale RO element, at different operating conditions, using parametric simulations based on Central Composite Designs. Outputs from this work not only provide insights into the microscopic flow characteristics of multiple full-scale elements in the RO pressure vessel, but also predicts the position where scaling would occur, at different feed conditions, for any RO products.

## 1. Introduction

Reverse osmosis (RO) is a process of separating solutes from liquid streams by applying external pressure to overcome the osmotic pressure of the solutes on the membrane, and has been widely used in desalination, pure water production, industrial wastewater treatment and water reclamation [1,2]. The water recovery level from brackish water desalination using RO processes can be up to 60%–95%, which is a critical operating parameter determining the cost effectiveness of the process. The higher the recovery, the higher the concentrations of inorganic salts that could potentially accumulate on the feed side of the membranes, leading to a more severe concentration polarization (CP) phenomenon and membrane scaling [3,4].

Coal mine waters usually contain complex inorganic salts with high total dissolved solids (TDS) and high hardness [5,6]. Most coal mine water also shows an alkaline pH [7]. The mixed effects of high ionic strength, high hardness and alkalinity, together with the effects of concentration polarisation, simultaneously affect the crystallisation and precipitation of sparingly soluble salts and scale formation on RO membranes, which constrains the operation of RO systems at high water recovery levels [8,9]. In addition, the treatment and recycling of coal mine waters often require high water recovery levels and “zero liquid discharge” (ZLD) options, which produce concentrated streams through the use of multi-stage RO systems, with a TDS of 80,000 mg/L brine sent to crystallisers [6]. The nature of the presented salts, as well as the requirements on high water recovery and ZLD, have contributed to the current technical challenges of the coal mine water treatment and recycling industry.

The current method of designing spiral-wound RO systems relies on commercial design packages such as WAVE (Dupont Water Solutions, USA), IMSDesign (Nitto) and Winflows (Suez) from membrane suppliers. However, as these commercial packages apply “black-box” models, that rely on empirical correlations obtained through experimental measurements using a specific membrane product and trial-and-error methods, it is difficult for the end user to assess the likelihood of which RO membranes in the whole process would be exposed to higher scaling potential at different operating conditions. There have been studies on the use of design software and system design tools on RO systems. Alhadidi et al. [10] used commercially available RO design software programs such as WAVE (in its previous version known as ROSA), IMSDesign, 4Aqua and Phreeqc, as well as manual calculations using the ASTM method, to calculate the scaling potential of various sparingly soluble compounds in a full-scale RO plant. The results showed that different scaling potentials were obtained for the same feedwater when different design software was used. Song et al. [11] developed a 2D mass transfer model to simulate a two-stage full-scale RO process. The main model outputs are water recovery levels, pressure drop and permeate TDSs, which showed good agreement with the operational data from a real plant. Fouling/scaling, however, was not considered in this model. Ruiz-García et al. [12] developed a computational tool that can predict the maximum water recovery without sparingly soluble salts formation. The algorithm developed in this work was validated using the experimental data of four full-scale BWRO desalination plants, proving that the proposed program is closer to actual data than the results predicted by the computational tool supplied by the manufacturer. These process modelling studies used 2D mass transfer equations and did not consider the variation of flow behaviour and mass transport caused by spacer-filled channels in the real spiral-wound modules.

Computational fluid dynamics (CFD) packages have been used to simulate 2D, 3D, unsteady and steady flows in RO modules [13,14,15,16,17,18,19,20]. Many models provide insights into the flow behaviour in spacer-filled channels, which can be used to understand mass transfer and concentration polarisation phenomena and to improve the design of the feed channel of RO membrane cells [21,22,23,24,25,26,27,28,29]. The limitations are that these simulations often neglect water permeation and are lacking information on the predictions of the fouling/scaling behaviour of full-scale RO modules and full RO trains. As such, the current design of RO plants still relies on the use of commercial packages supplied by membrane manufactures [30]. Ruiz-García and Nuez have developed a 2D mathematical model to simulate the impact of different feed spacers on water recovery, permeate salt concentration and the specific energy consumption of full-scale BWRO and seawater RO processes [31,32]. These studies enabled simulations of RO processes with novel spacer designs; however, the model does not include the prediction of scaling by sparingly soluble salts. Unlike CFD simulations, it cannot be used to provide spatial information on concentration polarisation and scale formation in a RO process train. Karabelas et al. [33] developed a model to describe the spatial evolution of scaling, but the model is based on the empirical correlation of the scale-mass deposition rate and the supersaturation ratio. The accurate prediction of the location where scaling occurs, using numerical methods rather than relying on empirical correlations, is particularly important for the coal mine industry, which requires multi-stage RO systems to treat water with high TDSs, hardness and alkalinity. The visualisation and prediction of the spatial distribution of scale formation can be used to optimise the addition of antiscalants.

In this study, we developed a 3D numerical model to predict the scaling behaviour of calcium sulphate in a full-size six-element RO pressure vessel treating coal mine water. The water production and scaling potential of calcium sulphate was computed by CFD in each RO element. Parametric simulations were also applied to study a wide range of operating variables, including feed pressure, flowrate, feed TDS concentrations and calcium sulphate concentrations. The spatial distribution of salts on membrane surfaces in the six-element RO train, and the location of the appearance of calcium sulphate scaling at different operating conditions, can be predicted.

## 2. Materials and Methods

### 2.1. The Coal Mine Water and Current Treatment Process

**Figure 1:**
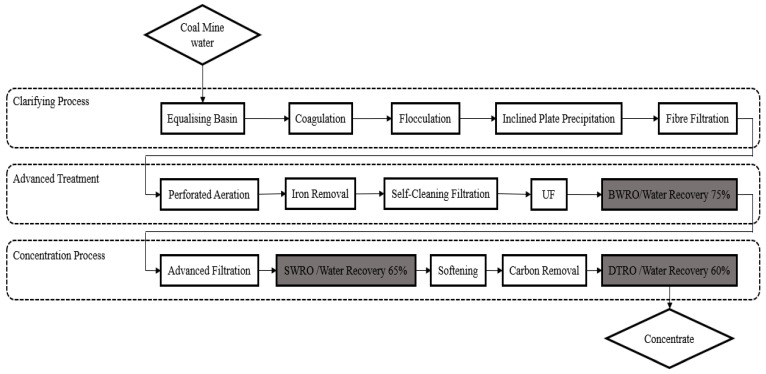
Process flow diagram of coal mine water desalination plant at Hongqinghe Coal Mine.

A multi-stage RO system with a production of 600 m^3^/h was designed for Hongqinghe Coal Mine, located in Ordos City, Inner Mongolia. The complete water treatment process contains a clarifying process, an advanced treatment process with ultrafiltration (UF) membranes, brackish water reverse osmosis (BWRO) systems and a brine treatment process for ZLD (Figure 1). The effluent produced from the BWRO unit is recycled and reused for the mine site. Brine from the BWRO unit is further treated by a seawater reverse osmosis membrane (SWRO) unit (Figure S1). The high salinity concentrate produced by the SWRO unit is fed to the third stage of the disk and tube reverse osmosis (DTRO) membrane unit. The highly concentrated brine enters the downstream crystallization process to achieve ZLD (the evaporation crystallization process section is not shown in Figure 1). The treated water produced by the SWRO and DTRO units in the concentration process is also used as domestic water for the mine site.

The whole desalination system includes three RO systems, which are represented by the grey boxes shown in Figure 1. The designed water recovery levels for the BWRO, SWRO and DTRO is 75%, 65% and 60%, respectively. The TDSs of the feedwater was 2610 mg/L with an alkaline pH of 8.2. The main compositions of the feedwater are shown in Table 1.

### 2.2. The BWRO Unit

**Figure 2:**
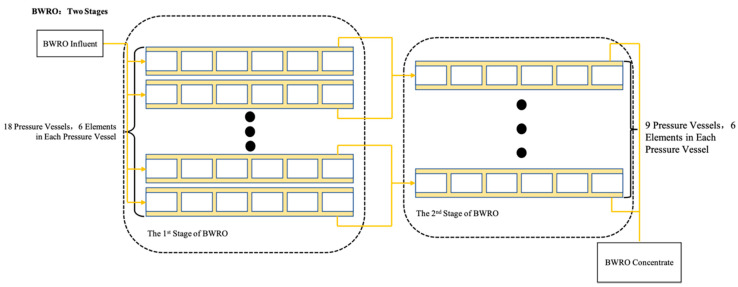
BWRO membrane assembly arrangement.

The BWRO unit in the plant has four parallel trains of two-stage BWRO systems. The first stage in each train contains 18 parallel pressure vessels, and the second stage contains 9 parallel pressure vessels (Figure 2). Each pressure vessel contains 6 membrane elements (Dupont FilmTec BW30-400).

### 2.3. CFD Model Setup

A three-dimensional model was developed to simulate the full-sized spiral-wound RO membrane module (Dupont FilmTec BW30-400) using a commercial CFD package Ansys CFX (2019R3) (Ansys Inc., USA). The methods of geometry (the Archimedean spiral) development and mesh creation can be found in our previously published work [34]. Transport of the solute (salt) at both the feed and the permeate sides was calculated using the species mass fraction transport equation:

(1) $$\frac{\partial }{\partial t}\left(\rho C\right)+\frac{\partial }{\partial x_{i}}\left(\rho u_{i}C\right)=\frac{\partial }{\partial x_{i}}\left(\rho D\frac{\partial C}{\partial x_{i}}\right)+S_{i}$$

where *C* represents the mass fraction of the solute, *u_i_* and *x_i_* represent the flow velocity and distance in the *i*th direction. *D* is the diffusion coefficient (1.33 × 10^−9^ m^2^/s for Na^+^ ions, 1.38 × 10^−9^ for Cl^−^ ions, 7.93 × 10^−10^ m^2^/s for Ca^2+^ ions, 7.05 × 10^−10^ for Mg^2+^ ions and 1.07 × 10^−9^ for SO_4_^2−^ ions). *S_i_* represents a source term.

The solvent (water) and solute (ions) transported through the RO membranes were simulated by adding source terms to the Continuity equation and the species mass fraction transport equation (Equation (1)), respectively. On the membrane surface:

(2) $$S_{s}=-\frac{J_{s}\cdot a}{V}$$

where *J_s_* is the flux of solute (ion) *j*; *a* is the effective membrane area; *V* is the corresponding volume in the computational domain.

The negative source terms for solvent and solute were determined using Equations (3) and (4):

(3) $$\;J_{w}=A\left(25\;{}^{\circ}C\right)\times TCFA\times \left(∆P-∆\pi \right)$$

(4) $$J_{s}=J_{w}\times C_{p}=B\left(25\;{}^{\circ}C\right)\times TCFB\times \left(C_{m}-C_{p}\right)$$

where $\;J_{w}$ is the water flux through the membrane; *A* (9.56 × 10^−1^^2^ m/s/Pa, Dupont published data for Dupont Filmtec BW30-400) and *B* (5.58 × 10^−8^ m/s,) are the water permeability coefficient and salt permeability coefficient at 25 °C, respectively; *TCFA* and *TCFB* are the temperature correction factors for *A* and *B; DP* is the pressure difference at the two sides of the RO membrane; Dp is the osmotic pressure; *C_p_* and *C_m_* represent the ion concentrations in the permeate and on the membrane surface, respectively.

The k-ω turbulence model was employed to simulate the bulk flow which was in the laminar turbulent transition zone. The membrane surface was set as a permeable membrane. The surface of the feed spacer was set as a non-slip boundary [35,36]. A high-resolution scheme was used to solve for the fluid flow equations. All simulations were performed as steady-state simulations, with the convergence target of 10^−4^ achieved for the pressure and the flow velocities, and 10^−6^ obtained for the mass fraction of ions. The flow velocity, pressure and concentration of ion species, calculated from the outlet of the precedent element, were used as the inlet conditions to the successor element.

In the current simulations, the feedwater to BWRO was set to contain five inorganic ions, Ca^2+^, Mg^2+^, Na^+^, SO_4_^2−^, and Cl^−^, with a concentration of 31.1 mg/L, 8.9 mg/L, 881 mg/L, 1030 mg/L and 679.5 mg/L, respectively. Carbonate species can also lead to the scaling of calcium carbonate; however, it is easier to manage than calcium sulphate scales, as the concentration of carbonate and saturation of CaCO_3_ is pH dependent. Given that acid dosing is widely used in RO treatment plants [37,38,39], and the complexity of carbonate species chemistry (from an open system to a closed system, i.e., the pressure vessels), the current work focusses on the simulation of calcium sulphate scaling. The feed flowrate, permeate flowrate, and feed TDSs of each element, as computed by CFD, was compared with the results calculated from the commercial RO design software WAVE (Dupont, USA).

### 2.4. Simulation of Scaling Potential on the First-Stage BWRO

The scaling potential of the sparingly soluble salt CaSO_4_ (in its likely format of gypsum) on the membrane surface was computed by calculating calcium ion and sulphate ion concentrations at specific locations on the membrane surface using CFD, for different membrane elements with varying feed pressure and feed concentrations from the lead element to the tail element in the first stage of the BWRO process. The Scaling Index (SI) was used to calculate gypsum scale formation (Equation (5)) [40,41]:

(5) $$SI=\log\left(\frac{\left\{{\mathrm{Ca}}^{2+}\right\}\cdot \left\{{\mathrm{SO}}_{4}^{2-}\right\}}{K_{sp}}\right)$$

where {Ca^2+^} and {SO_4_^2−^} are the activities of calcium and sulphate ions on the membrane surface, respectively, and *K_sp_* is the solubility product of the solutions with respect to gypsum, calculated using Visual Minteq. The activities of calcium and sulphate ions on the membrane surface were calculated by applying the activity coefficients of ions to the concentrations of ions on the membrane surfaces calculated by CFD. The activity coefficients were calculated using the Pitzer model that considers the effect of ionic strength, the electrostatic effects and short- and long-range ion interaction forces for salinity, up to 6 M [42,43].

### 2.5. Prediction of CaSO_4_ Scaling Threshold Conditions

CFD simulations were performed for a wide range of conditions with four operating variables: calcium sulphate concentration in the feed, concentrations of total dissolved solids (TDS), feed pressure and feed flow velocity. The range of each variable is listed in Table 2. Central Composite Designs (CCDs), also known as Box–Wilson designs, were used to create a matrix of simulation conditions [44]. A total number of 24 simulations were performed through Ansys Parametric Studies (Ansys Inc., USA). Since the output from each simulation variable was continuously analysed, it could be used to predict the appearance of calcium sulphate scaling at different conditions and different locations on the membrane module.

## 3. Results and Discussion

### 3.1. CFD Simulation Results on the First Stage of BWRO

**Figure 3:**
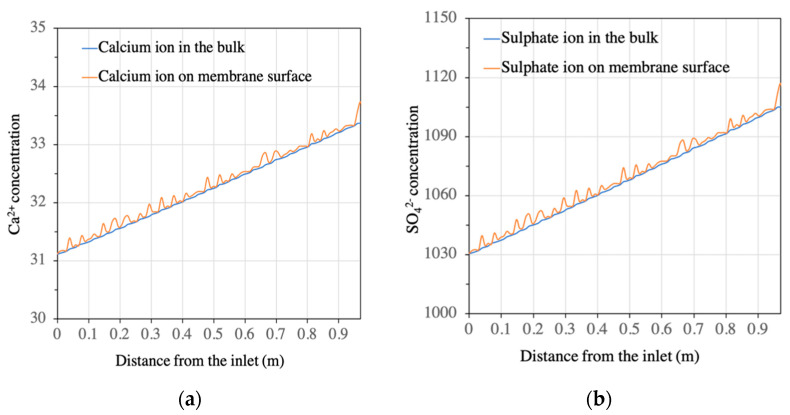
Calcium and sulphate ion concentrations in both bulk solution and on membrane surface in the 1st element of the 1st stage: (**a**) calcium ion; (**b**) sulphate ion.

**Figure 4:**
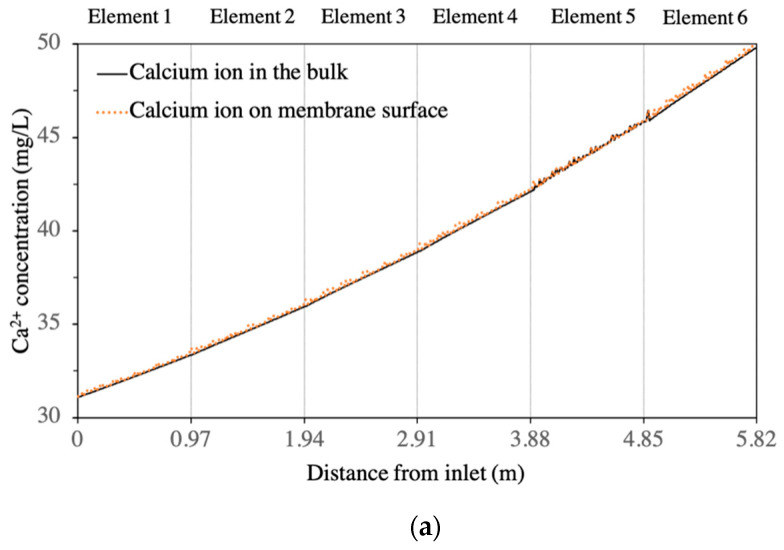

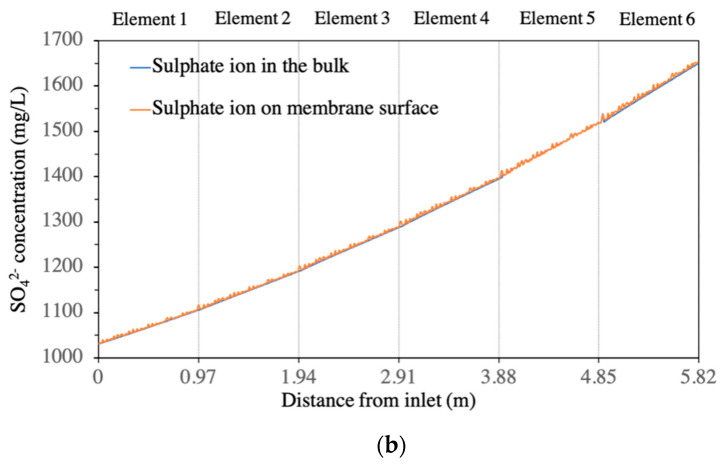
Calcium and sulphate ion concentrations in both bulk solution and membrane surface of the 6 elements in the 1st stage: (**a**) calcium ion; (**b**) sulphate ion.

CFD simulations were performed on each of the BW30-400 RO elements in the first stage of the BWRO process (i.e., the whole pressure vessel containing six elements). A quantitative analysis showed that the presence of the feed spacer led to a periodic fluctuation of salt concentration (Ca^2+^ and SO_4_^2−^) in the boundary layer of the membrane surface from the lead element to the tail element of the pressure vessel (Figure 3 and Figure 4). The concentration of calcium increased from 31.1 mg/L at the feed inlet to 49.9 mg/L on the membrane surface at the outlet position on the tail element, while the sulphate ion concentration increased from 1030 mg/L at the feed inlet to 1654 mg/L at the outlet position on the tail element. This change corresponds to an increase in SI from −1.22 to −0.96, indicating that calcium sulphate will not form a scale in the first stage of BWRO with the current design. This aligned with the results predicted by the commercial design software WAVE (Dupont, USA).

The CFD-simulated feed pressure, feed flowrate to each RO element and permeate flowrate generated by each RO element were compared with those predicted by WAVE (Dupont, USA) (Table 3). The results showed that CFD predicted a slightly higher pressure loss for each element. The difference between CFD-simulated feed pressure and values provided by WAVE increases from 0.7% for Element 2 to 3.3% for Element 6. The overall water recovery for Stage 1 of BWRO predicted by CFD is 55% compared to 57% by WAVE, with the largest difference of 1.2% occurring in Element 3. The maximum difference between CFD-simulated permeate flowrate and WAVE-predicted values is less than 10%. The difference between CFD-simulated values and those projected by WAVE could be due to the following reasons. Firstly, in the current CFD simulations, we used a constant permeate salt concentration (*c_p_*) measured from a lab scale crossflow RO cell, which could be different from the values used in WAVE. Permeate concentrations affect the mass sink term in Equation (2) and consequently the feed flow velocity (feed flowrate) and permeate flowrate. Secondly, WAVE used an average concentration polarization factor which is an empirical correlation derived from the average element recovery. Thirdly, the concentration (feed) side pressure drop calculated in WAVE is also based on an empirical correlation. The solutions from WAVE depend on these correlations, which were derived using a simple arithmetic average of the inlet and outlet conditions. Given that the outlet conditions are often unknown, WAVE uses iterative trial and error to achieve the final solution [37]. In contrast, CFD computes local ion transport numerically, which does not rely on empirical correlations developed from experimental measurements at certain conditions and trial-and-error methods. Thus, outputs from CFD are numerical results rather than results calibrated from experimental measurements at certain conditions. As a numerical tool, the error of CFD simulations can be attributed to discrepancy between the 3D geometry of the spacer and the characteristics of the real spacers, as well as the mathematical error caused by turbulence models. However, overall, the consistency between CFD-simulated results and values calculated by WAVE suggested good accuracy of the current CFD model. Further proof of the accuracy of CFD can be achieved through simulating different full-scale RO modules from other suppliers and comparing them with the corresponding commercial design software, as well as with experimental data from a real plant operated at well-controlled conditions.

### 3.2. Prediction of the Threshold of Calcium Sulphate Scaling at Different Operating Conditions

**Figure 5:**
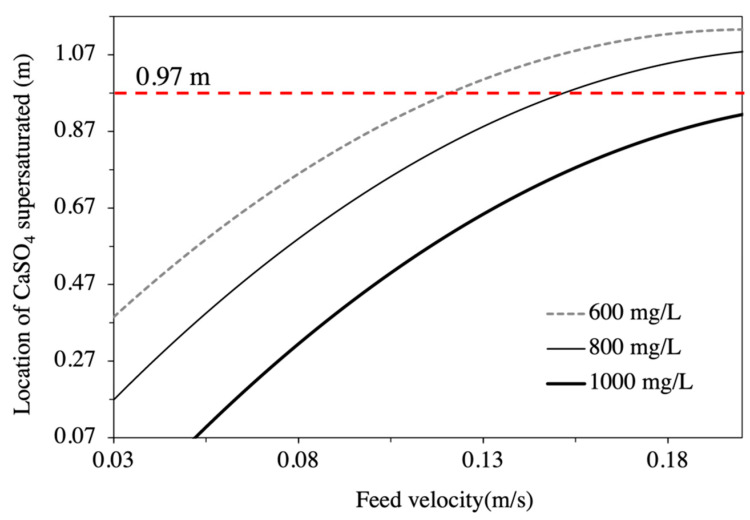
Position on RO element where CaSO_4_ supersaturated at different feed conditions.

A total of 24 sets of CFD simulations were conducted to predict the threshold of calcium sulphate scaling potential at different operating conditions. As coal mine water often contains high TDS, the current study covers a wide range of TDS up to 25,000 mg/L. Figure 5 shows the position on the BW30-400 RO element with calcium sulphate being supersaturated for feedwater containing 10,000 mg/L of TDS and with a feed pressure of 15.16 bar. It can be seen that, with different initial calcium sulphate concentrations and feed velocities, the location on the membrane where calcium sulphate becomes supersaturated varies. With the initial calcium sulphate concentration of 600 mg/L, scale might form at 0.37 m from the inlet to the RO element at a feed velocity of 0.03 m/s. For the same concentration (600 mg/L) of calcium sulphate in the feedwater, if the feed velocity is increased to above 0.12 m/s, scale will not occur in the first RO element (up to 0.97 m). However, when the initial concentration of calcium sulphate in the feedwater is increased to 800 mg/L, the feed velocity needs to increase to be above 0.15 m/s to avoid the scaling of calcium sulphate in the first element. When the initial calcium sulphate concentration is 1000 mg/L, scaling cannot be avoided under the simulated condition.

It should be noted that these results are based on the linear regression of the outputs from the 24 sets of simulations. A more sophisticated approach, that considers the practical operational aspects of the RO process (e.g., data that do not have a practical meaning should not be included during the DOE), should be taken to further improve the accuracy of this model. This is a subject of ongoing work. Future work also includes the integration of carbonate speciation into CFD models that can predict the scaling of both calcium sulphate and calcium carbonate.

## 4. Conclusions

This work developed a 3D CFD model to simulate the flow behaviour of a six-element RO pressure vessel. The model can be used to quantify and visualise the spatial distributions of salt concentrations on RO membranes, and predict the occurrence of calcium sulphate scaling. This model provides more flexibility for the optimisation of RO configurations, as the designers can obtain information on the exact location of the occurrence of scaling, rather than receiving a generic warning that scaling may occur from the current widely used commercial RO design software. It can also help reduce the operating costs by having a more flexible scaling mitigation approach. Antiscalants can be added at the location where scaling would occur, rather than simply being added to the feedwater. It also helps to identify the location where a turbulence promoter could be introduced to reduce scaling potential. As the CFD model can predict the performance of any full-scale spiral-wound element, and does not rely on the use of existing software provided by membrane manufacturers, the barrier of using novel RO membranes that are different from commercially available products is reduced. This work represents the first step of using CFD tools to assess and predict the spatial information of scale formation in full-scale RO processes. Although the current work has included the effect of concentration polarisation and ionic strength on calcium sulphate scale formation, it only considered the precipitation of a single sparingly soluble salt. Future work will include the coupling of a chemical speciation model, that involves the interactions of different sparingly soluble salts, with hydrodynamic modelling. Variations of pH on the membrane surface can also be modelled through this integration process.

## Figures and Tables

**Table 1:** Inorganic salt compositions of feedwater to BWRO unit in the Hongqinghe coal mine water treatment plant.

Test Parameters	Unit	Feed
pH	mg/L	8.2
TDS	mg/L	2610
Alkalinity	as CaCO_3_ mg/L	308.5
Hardness	as CaCO_3_ mg/L	109.5
Ca^2+^	mg/L	31.1
Mg^2+^	mg/L	8.9
Fe^2+^	mg/L	0.08
Al^3+^	mg/L	0.22
Ba^2+^	mg/L	0.018
K ^+^	mg/L	10.2
Na^+^	mg/L	881
Cl^-^	mg/L	393
F^-^	mg/L	3.8
SO_4_^2-^	mg/L	1030
NO_3_^-^	mg/L	0.31
SiO_2_	mg/L	3.41

**Table 2:** Range of operating variables used in CFD simulations.

**Variable**	**Range**
CaSO_4_ (mg/L)	25–1325
TDS (mg/L)	1000–25,000
Feed pressure (bar)	10.84–28.12
Feed flow velocity (m/s)	0.03–0.27

**Table 3:** Comparison of results obtained from CFD and WAVE.

Element Number	CFD-Simulated Results			WAVE-Simulated Results			Difference in Permeate Flowrate (%)
	Feed Pressure (Bar)	Recovery (%)	Permeate Flowrate (m³/h)	Feed Pressure (Bar)	Recovery (%)	Permeate Flowrate (m³/h)	
1	9.46	10.4	0.87	9.46	11.5	0.96	9.37%
2	9.14	11.1	0.83	9.21	12.2	0.90	7.78%
3	8.87	11.7	0.78	8.99	12.9	0.84	7.14%
4	8.63	12.8	0.75	8.81	13.7	0.77	2.60%
5	8.43	13.8	0.71	8.66	14.4	0.70	1.43%
6	8.25	15.4	0.68	8.53	15.1	0.63	7.94%

## Supplementary Materials

The following are available online at https://www.mdpi.com/article/10.3390/membranes11070521/s1, Figure S1: flow process diagram of BWRO and SWRO processes designed for the coal mine site.
