# Supplementary figures and images for: Numerical Simulations of Calcium Sulphate Scaling in Full-Scale Brackish Water Reverse Osmosis Pressure Vessels Using Computational Fluid Dynamics
Source: Membranes (Basel). 2021 Jul 11;11(7):521. doi: 10.3390/membranes11070521 (PMC8306596; doi:10.3390/membranes11070521)

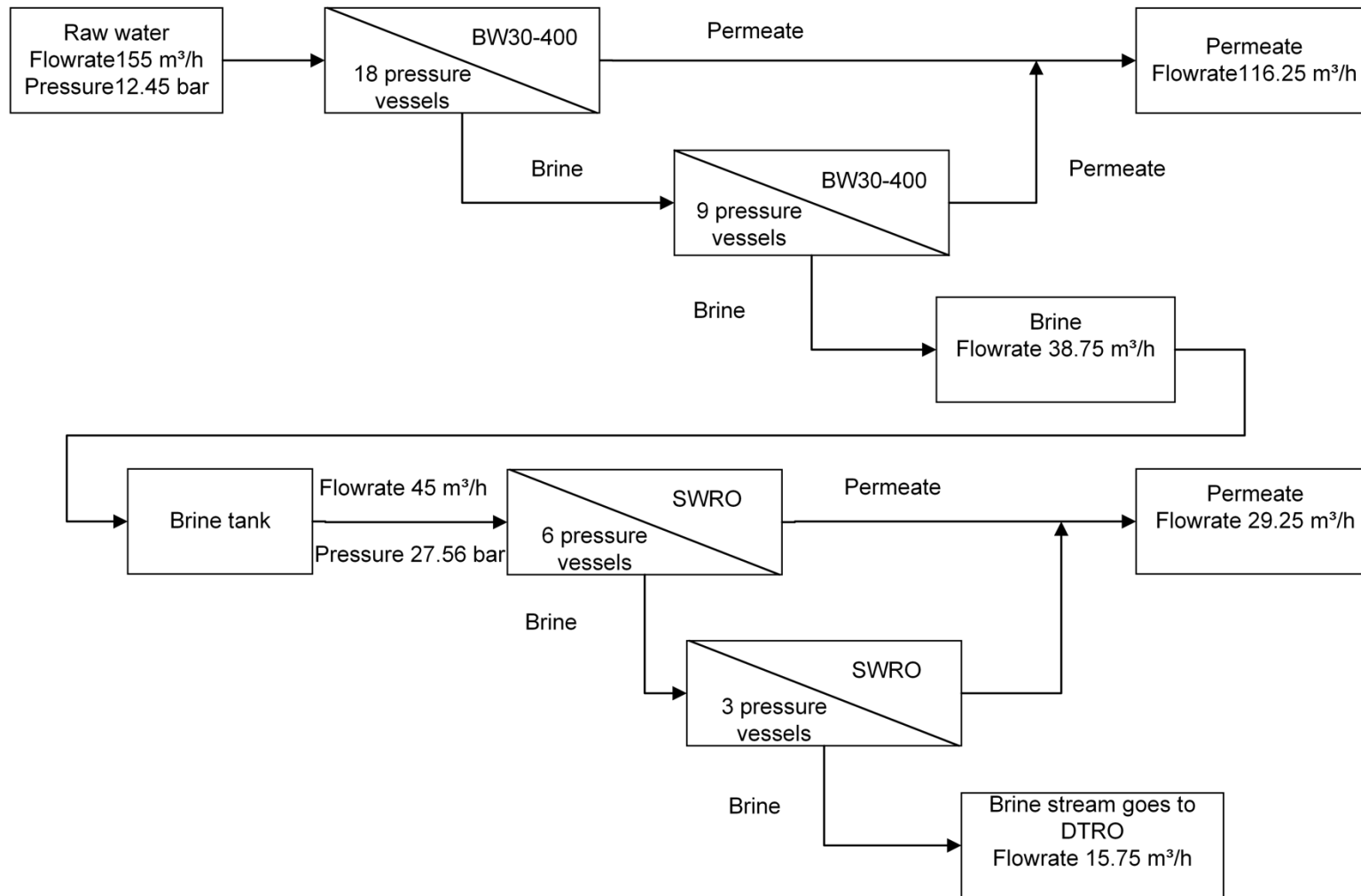

Figure S1 Flow process diagram of BWRO and SWRO processes designed for the coal mine site

Supplement: Supplementary file 1 [file membranes-11-00521-s001.zip › membranes-1260604-supplementary.pdf]
